# Accelerating the Layup Sequences Design of Composite Laminates via Theory-Guided Machine Learning Models

**DOI:** 10.3390/polym14153229

**Published:** 2022-08-08

**Authors:** Zhenhao Liao, Cheng Qiu, Jun Yang, Jinglei Yang, Lei Yang

**Affiliations:** 1 Department of Civil Engineering, College of Civil and Transportation Engineering, Shenzhen University, Shenzhen 518060, China; 2 Institute of Mechanics, Chinese Academy of Sciences, Beijing 100190, China; 3 Department of Mechanical and Aerospace Engineering, Hong Kong University of Science and Technology, Hong Kong 999077, China; 4 China Railway 5th Bureau Construction Engineering Co., Ltd., Guizhou 550081, China; 5 HKUST Shenzhen-Hong Kong Collaborative Innovation Research Institute, Shenzhen 518031, China; 6 Foshan SMN Materials Tech Co., Ltd., Nanhai District, Foshan 528200, China

**Keywords:** composite laminate, mechanical property, layup design, finite element simulation, neural network

## Abstract

Experimental and numerical investigations are presented for a theory-guided machine learning (ML) model that combines the Hashin failure theory (HFT) and the classical lamination theory (CLT) to optimize and accelerate the design of composite laminates. A finite element simulation with the incorporation of the HFT and CLT were used to generate the training dataset. Instead of directly mapping the relationship between the ply angles of the laminate and its strength and stiffness, a multi-layer interconnected neural network (NN) system was built following the logical sequence of composite theories. With the forward prediction by the NN system and the inverse optimization by genetic algorithm (GA), a benchmark case of designing a composite tube subjected to the combined loads of bending and torsion was studied. The ML models successfully provided the optimal layup sequences and the required fiber modulus according to the preset design targets. Additionally, it shows that the machine learning models, with the guidance of composite theories, realize a faster optimization process and requires less training data than models with direct simple NNs. Such results imply the importance of domain knowledge in helping improve the ML applications in engineering problems.

## 1. Introduction

Fiber-reinforced composites are considered as important materials in various engineering applications as they are superior materials possessing high specific strength, high specific modulus, light weight, and good resistance to aging and corrosion. With the increasing demand for high-performance composite materials, advanced fibers, matrices, and their composites have been developed with remarkable mechanical or non-mechanical properties [1,2,3,4]. After impregnating the fibers with a matrix to form a single ply, the ply is stacked in specific directions to meet the design requirements. Such laminated structures are widely used in the civil and aerospace engineering fields. The anisotropic characteristics of composite laminates provide wide flexibility in their load-carrying capacity, which has promoted the development of a proper design methodology for laminates [5,6]. Many researchers have recently started to investigate the layup design of composite laminates. For example, Kharghani [7] used numerical stacking optimization to reduce the free edge effect around the composite plate hole, and Maung [8] completed the Wageningen B of a series of marine propellers. To optimize the ply design, Abdallah [9] evaluated the ply angle and stacking sequence using LS-DYNA finite element software and discovered that the optimized blade design with a curved fiber stack resulted in a 20% reduction in Tsai–Hill failure index under the same pitch change. Tensile and radial compressive loads affect the mechanical properties of glass/phenolic composite tubes. Nebe [10] investigated the effect of stacking sequence and circumferential layer drop position on the mechanical response of an internal-pressured type IV composite pressure vessel and the results indicated that the mechanical properties of the laminate were significantly influenced by the laminate design. At the same time, the difficulty in studying laminate design is that there is no universal optimal solution for the best laminate design under different working conditions. A method for optimizing the layup design applicable to various operating conditions awaits to be explored.

In recent years, with the rapid advancement of machine learning (ML) algorithms and accessible open-source libraries, data-driven methods have frequently been utilized for efficient and effective structural design and characterization including composite laminate structures [11]. For example, Wanigasekara [12] established an automated fiber placement unidirectional composite laminate prediction model using artificial neural networks (ANN) to estimate the quality and integrity of the manufactured laminate. Bharata [13] combined the finite element method (FEM) and ML to analyze the buckling of an inclined laminated composite plate. Qiu [14] proposed a novel characterization method for composite fracture toughness using ML to extract information from the indirect measurement data. Erban [15] adopted ML technology to accelerate the design process of composites by replacing the time-consuming FEM analysis. Veivers [16] used particle swarm optimization (PSO) to optimize the layup design of generic tubular geometries under simultaneous thermal and mechanical loading conditions, and Cai [17] studied the application of ML methods to analyze the dynamic strength of 3D-printed polypropylene (PP) composites. A number of results in the literature show the successful application of machine learning in composite materials, which provides a good idea for the integration of machine learning and composites.

Even though ML performs well in terms of predicting the accuracy for complex nonlinear problems and is efficient in real-time evaluation, it requires the input of large amounts of data, which is a vexing problem. The easiest way to obtain training data is through numerical simulations, but a reliable and low-cost simulation technique should be developed. An alternative method of acquiring data can be from the literature or experiments, which are exhausting work. To alleviate the problems related to data, a variety of methods have been adopted by many scholars. Data augmentation [18], which artificially generates training data, helps expand the available data samples. Specialized learning algorithms [19] such as transfer learning, which transfer knowledge from the domain where training data are abundant to the target domain where data are scarce. Model architecture design [20], which improves the prediction accuracy with limited training data by constraining the parameter space with prior knowledge. For the application of ML to engineering problems, researchers have established physical-informed neural network (PINN) [21] or theory-guided machine learning (TGML) [22], which focus on how to import the corresponding domain knowledge to help the ML model better extract the physical laws hidden behind less training data. Theoretical guidance is implemented by employing pre-known governing equations as constraints or logically designing the model architecture according to the related theories [21,23,24]. Therefore, a combination of the theory of composite materials with machine learning is considered to address the issue that machine learning requires a large dataset.

Designing composite laminates is not an easy task. On one hand, the multi-scale feature of the composite shows complex behavior, indicating difficulty in the forward evaluating structural response [24]. On the other hand, multiple design variables such as constitutive materials, microstructure, and layups need to be determined, therefore requiring time-consuming inverse optimization [25]. The use of an appropriate neural network model and optimization algorithm can significantly reduce the fitting and optimization time. However, according to the current literature, most of the time, the usage of the ML-based design method lies in the training data generation. Data generation accounts for a large portion of the overall optimization design time, which is a common problem in optimizing the composite performance using ML [26]. Our research aims to improve the efficiency of data utilization throughout the design process using the TGML model. Therefore, in this paper, we attempted to improve the performance of the model with the help of theoretical guidance using ML-based models with a few training data samples. For this purpose, we built a multi-layer interconnected neural network system following the logical sequences of composite theories regarding the stiffness and strength. With the forward prediction by the NN system and the inverse optimization by genetic algorithm (GA), the TGML model aims to provide optimum composite layup sequences under multiple design constraints.

In this paper, the TGML model was utilized for the efficient layup design of composite tubes with minimum deformation and maximum strength values under the loading conditions of torsion or bending. Section 2 describes the target problem and the model development in detail. In Section 3, the model performance and the effect of theory guidance are discussed. Our conclusions are presented in Section 4.

## 2. Materials and Methods

### 2.1. Problem Description

The design problems of composite tubes under bending or torsional loads were considered in this study. The design target was to provide the optimum layup sequences of the composite tube under the constraints of structural rigidity and strength. In this context, two types of design problems were investigated. One is the optimum layup sequences for maximizing the structural rigidity under the bending or torsional loads. The other is the optimum layup sequences that maximize the strength values. Several specific loading cases were investigated. A typical design scenario can be described as follows.

(a) The strength model considers two load conditions on the composite tube: under a bending stress of 1300 N and a torsional stress of 300 N · m. The corresponding layup sequences when the minimum failure indices are obtained are the optimal layup.
(b) In designing the stiffness model evaluation threshold, two design objectives were set considering that the stiffness of the laminate should be designed to meet the stiffness requirements in practical engineering applications:

1. Bending deformation stiffness greater than 250 N/mm
2. Torsional deformation stiffness greater than 1500 N · m/rad

The optimal solution was to use the smallest fiber modulus to meet these two stiffness conditions for the layup. In other words, the materials with the least fiber modulus were used to achieve the specified stiffness requirements by changing the layup.

The basic geometries of the tube were an inner diameter of 18 mm and the outer diameter was 23 mm and a length of 200 mm. The maximum number of layups was set to 16. The layup design followed the common guidelines of symmetry and balance and was chosen from (±θ[1]/±θ[2]/±θ[3]/±θ[4])s with *θ* in the range of [0, 90].

A hollow composite tube model was established using the Abaqus finite element software(Abaqus6.14, SIMULIA.Co., Ltd., Providence, RI, USA) to obtain a dataset for model training. Python scripts were written to generate the training data consisting of random fiber orientation module, random layups, and corresponding structural properties using the FEM model.

### 2.2. Materials

#### 2.2.1. Material and Fabrication

The HH-2082 unidirectional carbon fiber prepreg supplied by Henghai Technology of Dongguan, China was used. The material had a tensile modulus of 115 GPa. The preparation process was vacuum bag compression molding. The carbon fiber prepreg was laid on the aluminum tubes in a predetermined layup sequence after a uniform application of release agent on the surface of the 6061-grade aluminum tubes. In that order, the specimens should be wrapped using release cloth, film, the air felt, and a vacuum bag. The specimens were then heated to 95 °C in an OV301 oven (Easy Composites Co., Ltd., Beijing, China) connected to the vacuum pump, then cured for 6 h while the vacuum pump was constantly running to maintain a vacuum pressure for the test piece. After curing and cooling naturally, the aluminum tubes were removed from the carbon fiber round tube. After water cutting, we obtained the carbon fiber composite tube (the length was 200 mm, the inner diameter was 18 mm, and the outer diameter was 23 mm).

#### 2.2.2. Experimental Preparation

We prepared the composite tube according to the layup sequences output by the TGML model. Six specimens were prepared for each ply sequence, three of which were used for the torsion test and the other three for the bending test.

**Figure 1:**
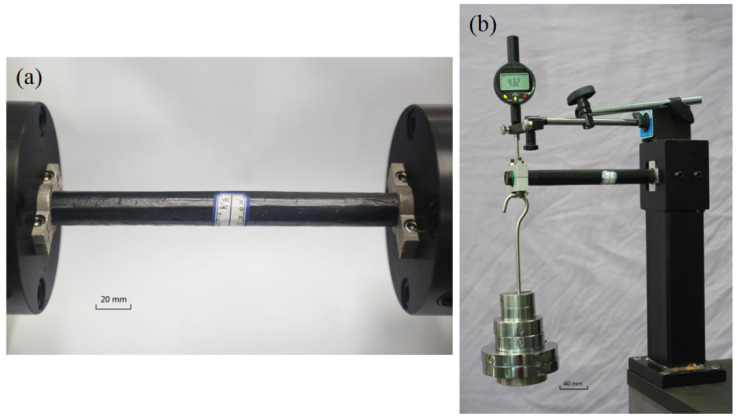
Test device. (**a**) Torsion test. (**b**) The cantilever beam test.

The torsion test was performed on a microcomputer-controlled torsion testing machine manufactured by Shenzhen Rigel Instrument Co., Ltd. With the model number RNJ500 and a maximum torque of 500 N · m and a torsional loading rate of 5 degrees per minute. When the torque reached its peak, it dropped by 30% to make the torsion experiment closer. A cantilever beam measured the bending stiffness by embedding one end of a circular pipe and applying a load to the other, with a displacement meter at the free end. Each time the weight was added, the value of the displacement meter was recorded. Before recording the value of the displacement meter, a prestress (850 g weight) was given to the composite tube, and a weight of about 1 kg was added each time. Figure 1b depicts the cantilever beam test.

### 2.3. Theory-Guided Machine Learning Layup Design Strategy

#### 2.3.1. TGML Model for Design with Optimum Strength

In this section, the design problem of finding layup sequences with optimum strength values is explored. Two loading conditions on the composite tubes were considered: bending and torsion. The design flowchart in Figure 1 indicates the problem of minimizing the failure indices for both load conditions. With a known layup sequence, the in-ply stresses of each layer in the laminate can be obtained by numerical simulation. The failure indices were then calculated using the composite failure theory of in-ply stresses as the input. Among the numerous theories on how fiber-reinforced polymer failure initiates and evolves, HFT, which can describe different failure modes, is one of the most widely used criteria in the field of composite modeling. Fiber failure criteria are incorporated to evaluate the laminate strength, as shown in Equations (1) and (2), where $X^{T}$, $X^{C}$, and $S^{L}$ are the longitudinal tensile, compressive, and shear strength, respectively.

Fiber tension ($\sigma_{11}>0$):

(1) $$f^{T}\left(\sigma \right)={\left(\frac{\sigma_{11}}{X^{T}}\right)}^{2}+{\left(\frac{\sigma_{12}}{S^{L}}\right)}^{2}$$

Fiber compression ($\sigma_{11}<0$):

(2) $$f^{\;C}\left(\sigma \right)={\left(\frac{\sigma_{11}}{X^{C}}\right)}^{2}$$

**Figure 2:**
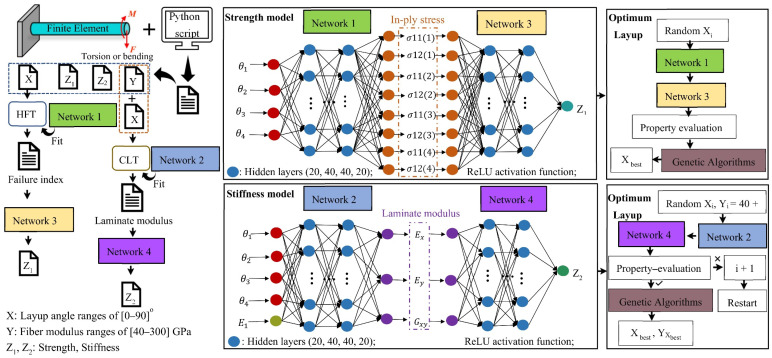
The theory-guided machine learning model process.

Following the flow chart, with NN instead of modules for numerical simulation and failure theory, Figure 2 shows the structure of the TGML model used to accelerate the layup design with the optimal strength values. Since the composite laminate was prescribed to be symmetrical and balanced with the layup of (±θ_1_/±θ_2_/±θ_3_/±θ_4_)s, a total of eight in-ply stresses in four angled plies were utilized as intermediate variables that connected Network 1 and Network 3. Then, the predicted failure indices were imported from the GA module to solve the minimization problem. It should be noted that instead of directly using the angle values as inputs, the corresponding cosine values were employed considering the mathematical formulation involved in the angles.

#### 2.3.2. TGML Model for Design with Optimum Stiffness

The design problem of finding layup sequences with the best stiffness values was explored. The fiber modulus started to increase from 40 GPa, and the layup sequences were randomly generated in the interval of [0–90]° with a step size of 5° degrees. These combinations were fed into the stiffness model as input signals. The above steps were repeated if the stiffness requirements were not met. If the stiffness design conditions were met, the output layup sequences combination was considered to be the best solution for the stiffness model. The laminar modulus in the four angular layers was the intermediate variable connecting the two neural networks. The fiber modulus minimization problems are depicted in Figure 2. The TGML model incorporates guidance from the CLT and uses a similar architecture to the strength model to establish the optimum stiffness. The GA module was applied to identify the best combination of layup sequences to satisfy the stiffness requirements.

CLT was used to obtain a homogeneous laminate stiffness including *E_x_*, *E_y_*, and *G_xy_*. First, Equation (3) helped to transform the stiffness coefficient matrix from a local fiber orientation to global coordinates including *E_x_*, *E_y_*, and *G_xy_*. First, Equation (3) helps to transform the stiffness coefficient matrix from a local fiber orientation to global coordinates. In Equation (3), $Q_{ij}$ is the single-ply stiffness coefficient in terms of local coordinates with a fiber angle of θ, while ${\bar{Q}}_{ij}$ is that in terms of the laminate coordinates. After combining the contributions of each layer, the equivalent stiffness matrix for the laminate can be obtained by Equation (4), where t is the laminate thickness consisting of n layers, and $t_{k}$ is the thickness of the single layer. With the equivalent stiffness matrix, the homogeneous modulus can be derived from the compliance matrix, which is the reciprocal of the stiffness matrix.

(3) $$\left\{\begin{array}{c} {\bar{Q}}_{11}=Q_{11}\cos^{4}\theta +2\left(Q_{12}+2Q_{66}\right)\sin^{2}\theta \cos^{2}\theta +Q_{22}\sin^{4}\theta \;\;\;\;\;\;\;\;\;\;\;\\ \;\;\;\;\;\;{\bar{Q}}_{12}=\left(Q_{11}+Q_{22}-4Q_{66}\right)\sin^{2}\theta \cos^{2}\theta +Q_{12}\left(\sin^{4}\theta +\cos^{4}\theta \right)\\ {\bar{Q}}_{22}=Q_{11}\sin^{4}\theta +2\left(Q_{12}+2Q_{66}\right)\sin^{2}\theta \cos^{2}\theta +Q_{22}\cos^{4}\theta \;\;\;\;\;\;\;\;\;\;\;\\ {\bar{Q}}_{16}=\left(Q_{11}-Q_{12}-2Q_{66}\right)\sin\theta \cos^{3}\theta +\left(Q_{12}-Q_{22}+2Q_{66}\right)\sin^{3}\theta \cos\theta \\ {\bar{Q}}_{26}=\left(Q_{11}-Q_{12}-2Q_{66}\right)\sin^{3}\theta \cos\theta +\left(Q_{12}-Q_{22}+2Q_{66}\right)\sin\theta \cos^{3}\theta \\ {\bar{Q}}_{66}=\left(Q_{11}+Q_{22}-2Q_{12}-2Q_{66}\right)\sin^{2}\theta \cos^{2}\theta +Q_{66}\left(\sin^{4}\theta +\cos^{4}\theta \right) \end{array}\right.$$

(4) $${Q^{\prime }}_{ij}=(\overset{n}{\underset{k=1}{\sum }}{\left(\;{\bar{Q}}_{ij}\right)}_{k}t_{k})/t$$

### 2.4. Model Main Parameter Settings

#### 2.4.1. Configuration of NNs

We used a feedforward neural network (MLPRegressor, MLP), which is the most common NN in practical applications. MLP can classify and regress nonlinear problems in composite materials. We chose the ReLU activation function as the activation function of the ML model, which can make the NN training faster and increase the network’s nonlinearity. The weight optimizer for NNs is “lbfgs”, and “lbfgs” is an optimizer in the family of quasi-Newton methods. For small datasets, “lbfgs” can make the model converge faster and work better. We set the hidden layer of the NNs to four layers. We set twenty, forty, forty, and twenty neurons in the four hidden layers, respectively, which we denoted as “hidden_layer_sizes = (20, 40, 40, 20)”. More hidden layers can bring more complex computing power, but too many hidden units will bring overfitting. We conducted much-debugging work in the early stage, and we found that when the hidden layer was set to four layers, it could help the TGML model to play well in regression performance. 

The number of neurons in the hidden layer has an impact on the performance of the NNs, and Equation (5) can help us find the appropriate number of hidden layer nodes, where *h* is the number of neurons in the hidden layer; *m* is the number of neurons in the input layer; *n* is the number of nodes in the output layer; and *a* is an adjustment constant between 1 and 10.

(5) $$h=\sqrt{m+n}+a$$

We tested and tuned the models many times. If training and generalization errors become high due to NN bias and underfitting, we gradually increased the number of hidden layer nodes until we obtained a satisfactory result.

#### 2.4.2. Configuration of the GA Module

We controlled the population generation based on the preset stiffness or strength constraint values. The genetic algorithm has four parameters that need to be set in advance, generally set within the following ranges:

(1) Group size: 20~100;
(2) The terminal evolution algebra of genetic algorithm: 100~500;
(3) Crossover probability: 0.4~0.99;
(4) Variation probability: 0.0001~0.1.

We set the population size to 100, the evolutionary termination generation of the genetic algorithm to 250, the mutation probability to 0.01, the crossover probability to 0.8, and the output precision to 5. By adjusting the parameters several times and comparing the optimization results, the above parameter settings of the genetic algorithm met the performance requirements of the model optimization.

## 3. Results and Discussion

To better observe the accelerating effect and the accuracy provided by the TGML models, we conducted research from two aspects. First, we analyzed the training performance of the TGML model, and the design scheme was generated accordingly. Second, the effect of the theory-guided model was investigated by comparing the performance of the TGML models trained on a small training set with direct NN systems trained on a large training set. The TGML models could achieve an accurate regression performance with a very small training set by designing efficient machine learning models that greatly reduced the overall machine learning time.

### 3.1. TGML Model Performances

It should be noted that all NNs in Figure 2 were the same, and the ANN configuration of the models without the theory-guided model were also the same. The model without theoretical guidance referred to the direct fitting and the regression of the training dataset derived from FEM. The layup sequence was the input to the without theory-guided models, and the output was the strength or stiffness result, without calculating the intermediate variables.

To realize the intelligent optimization design, “Network 1” took the layup as the input and the in-ply stresses as the output, which was a NN that fits the HFT. By connecting “Network 1” and “Network 3” serially, the entire strength TGML model could randomly generate a combination of layups that met the strength requirements and find the optimal global solution through the GA module. Similarly, in the stiffness TGML model, the inputs of “Network 2” were the layup, and the outputs were the laminate modulus, a NN that fits the CLT. The role of “Network 2” in the stiffness TGML model was similar to that of “Network 1” in the strength TGML models.

Each of these two problems generated 3000 random data points, which implies 3000 different layups and the corresponding properties. A total of 10% of these 3000 sets of data were utilized as the test dataset, which was used to test the regression performance of the models. The models were trained with 2700 data points. After training the models, the model output the predicted values with the test dataset as inputs, and a comparison was made between the predicted values and the output values of the test set. The training data for “Network 3” were obtained from the mathematical formulations of Equations (1) and (2) containing 2700 random inputs and the corresponding outputs. The construction process of the stiffness models was the same as that of the strength models. However, the training data of “Network 4” were obtained from the mathematical formulations of Equations (3) and (4).

**Figure 3:**
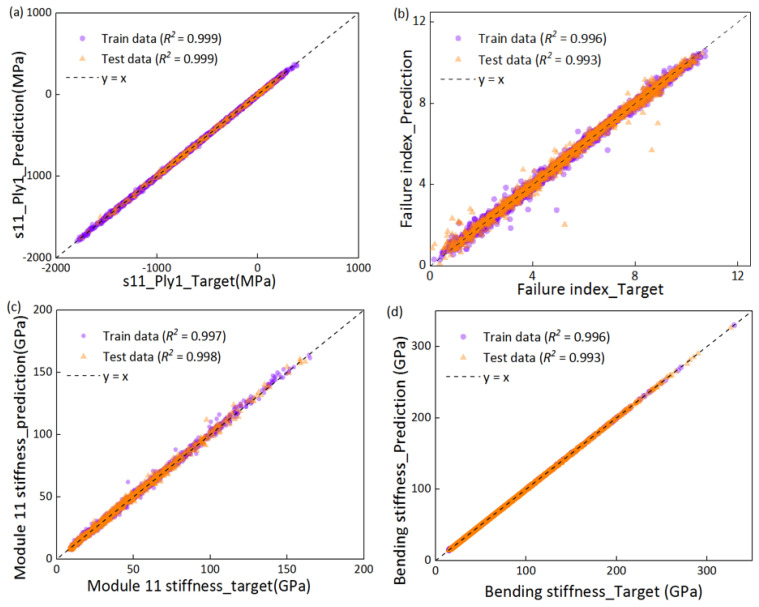
The regression performance for four networks in the proposed ML model. Models were trained with 2700 training data. (**a**) Network 1 for predicting the in-ply stresses S11 in four angled ply from the layup sequences. (**b**) Network 2 for predicting the Hashin fiber failure index from the in-ply stresses. (**c**) Network 3 for predicting the laminate modulus from four angle sequences and a single-layer board modulus. (**d**) Network 4 for predicting the stiffness from the laminate modulus.

After training, the predicted performance of the models is shown in Figure 3. Figure 3a,b shows the regression performance of “Network 1” and “Network 3”, respectively, which indicates the regression performance of the strength model. Similarly, the regression performance of the stiffness models is shown in Figure 3c,d. The coefficient of regression *R*^2^ > 0.95 demonstrates that the network models can accurately predict the properties, provided the material and layup are known. Therefore, the ML model was meticulously designed to generate data samples that would allow the network model to produce satisfactory results.

### 3.2. Effect of the Theory-Guide

**Figure 4:**
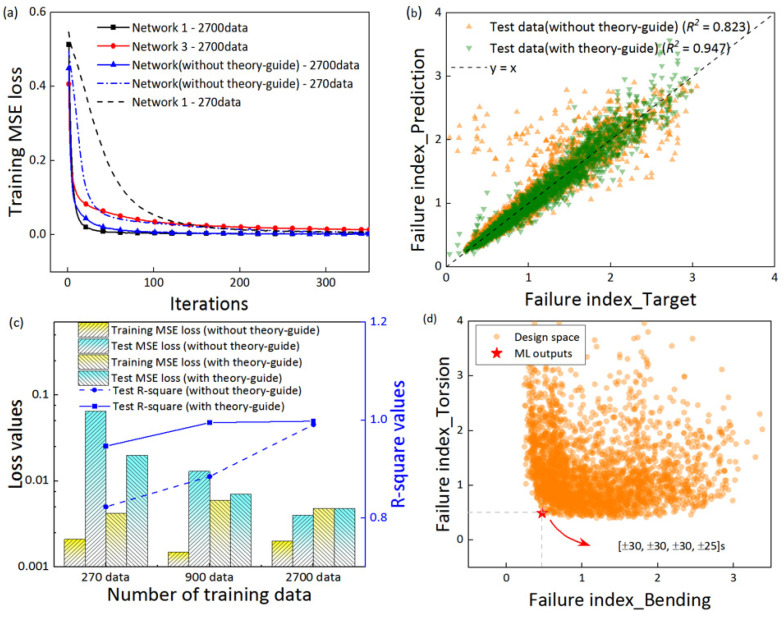
A comparison between the models with and without theory-guides under different amounts of training data. (**a**) Mean squared error on the 2700 and 270 training data samples with increasing iterations. (**b**) The regression performance on the test data samples when models were trained with only 270 data samples. (**c**) The loss values and *R*^2^ comparisons of the models trained with the 270, 900, and 2700 data samples. (**d**) The GA module searched for the optimal solution for 3000 sets of failure index solutions.

In this section, we compared the models with and without the theory guide at different amounts of training data. The models without the theory guide were directly trained using the layup sequences as inputs and the stiffness or strength as the outputs. According to the fitting performance, only the strength models are shown here due to the small difference between the stiffness and strength models. The comparison of the iteration convergence times of the mean squared error (MSE) values in Figure 4a showed that the difference between the models with under 2700 and 270 training data were marginal. The MSE iteration of the models with or without theory-guided both converged. However, for the training data, the TGML model fit better for the sample case with 300 data. Figure 4b reveals that under the 270 training dataset, the *R*^2^ value of the prediction performance of the strength TGML models was greater than that of the strength ML models. The regression performance of the TGML models was significantly better than the models without the theory-guided model.

As shown in Figure 4c, it can be seen that the TGML models exhibited different performances at the270, 900, and 2700 training sets, respectively. The TGML model presents a high regression performance and low loss values for both large (2700 data points) and small (270 data points) data volumes. The *R*^2^ values of the TGML models in 270 data points were similar to those of the *R*^2^ values with 2700 data points. Under the requirement of the regression performance of the models, the TGML models with 270 data points can be selected instead of those with 2700 data points for the optimal design of the layup sequences. However, the regression performance of the models without the theory-guided model under the different amounts of training data was significantly different. The MSE without the theory-guided model with 270 data points was more than ten times that of the without the theory-guided model with 2700 data points, indicating the poor prediction performance of the model without theoretical guidance in small data training. It is worth mentioning that the models with and without theoretical guidance exhibited higher *R*^2^ values and lower MSEs under training with 3000 datasets, thanks to the careful design of the NN configuration.

Combined with the optimization process in Figure 4d, it can be interpreted that the accuracy of the model is crucial in that the data points close to the optimal result are very dense. Otherwise, the optimal solution cannot be obtained. The GA module searches for the optimal layup solution, which is the solution closest to the origin on the diagonal of the coordinate axis, as shown in Figure 4d. The total design space is defined as all of the possible layup combinations with the angle increment of 1 degree. The optimal layup sequence was [±30/±30/±30/±25]s for the composite tube design problem. That is, the failure indicator reached a minimum value when the layup sequence was [±30/±30/±30/±25]s under a bending stress of 1300 N and a torsional stress of 300 N·m.

### 3.3. Calculation Efficiency

As can be seen in the figure above, the prediction performance of the TGML models with 270 and 2700 training sets was similar, which is another advantage of the TGML models. The TGML models still maintained the original regression performance despite greatly reducing the training datasets. The total computer run time spent training the TGML models on training sets with numbers of 270 and 2700 was compared in this study. The calculations were performed on a PC with an Intel i7-6700 CPU (Intel Co., Santa Clara, CA, USA). The total running time of the entire design process is listed in Table 1. The FEM generation data took up most of the time during the entire run. It took 135 min to calculate 2700 sets of data by finite element and 13.5 min to calculate 270 sets of data. The training of NNs did not take much time and completed the training of 2700 sets of data points in only 2 min. It took nearly 1.5 min to complete the training of 270 sets of data points. Obviously, the training time of the neural network and the amount of data have a nonlinear relationship. We predicted that the training time was not much different due to the small volume of training data. At the same time, the computing power of the computer will also affect the training time of the NNs. The ML models spent roughly 3 min in the optimization design stage with the GA module. Therefore, a significant reduction in data is a significant reduction in time cost. TGML is a path proposed to solve the shortcomings of traditional ML requiring a large amount of data.

### 3.4. Solutions Provided by the TGML Models

Some layup designs were generated through the TGML models, as shown in Table 2 and Table 3. The TGML models can be used to design layups with the highest strength or stiffness under different load conditions. The three cases in Table 2 represent the corresponding layups when the failure indices reached the minimum values under different load conditions. The three cases in Table 3 represent the layups with the lowest fiber modulus required to meet the different stiffness requirements. Table 2 and Table 3 show the optimal solutions under the corresponding conditions.

It is worth mentioning that for the layup design that satisfied the target stiffness in Table 3, the *E*_1*M*_ is the single-ply modulus in the fiber direction in the output of the TGML models. This is because of the combination of the CLT, but the strength calculation results did not yield the *E*_1*M*_. FEM further verified these results. The results were slightly different from those listed in the table, but the error was within an acceptable range. The experiments in the next section will further validate the results.

## 4. Experimental Validation

In this section, the composite tube specimens were prepared to use the optimized layup sequence produced by the TGML stiffness model, and two experiments of bending and torsion were conducted, respectively. Verification of the accuracy of the TGML model involves comparing the stiffness of the composite pipe under the specified laying sequence to the stiffness produced by the model. The layup sequences used were the results of the stiffness models shown in Table 3. Three specimens were tested for each ply combination.

The output of the stiffness TGML model from Table 3 revealed that the layup sequences and fiber orientation modulus were included. However, a fixed fiber direction modulus *E*_1*T*_ = 115 GPa was used in the experiments. Therefore, the dimensionless stiffness to the modulus ratio was utilized for evaluation. 

**Figure 5:**
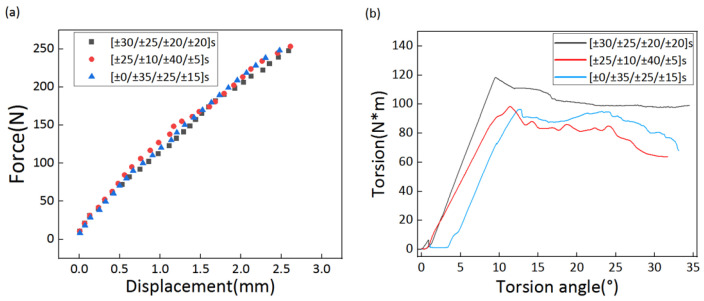
The experimental record curve. (**a**) The force–displacement curves for the three composite tubes under the cantilever bending test. (**b**) The torsion–torsion angle curves for the torsion test.

Three layup sequences were used for the experiments, which were the combination of [±0/±35/±25/±15]s and *E*_1*T*_; [±25/±10/±40/±5]s and *E*_1*T*_; and [±30/±25/±20/±20]s and *E*_1*T*_. Figure 5 displays the torsion–torsion angle curves for the torsion test as well as the force–displacement curves for the three composite tubes under the cantilever bending test. The stiffness can be obtained by simply calculating the curvature of the curve in the elastic phase. The torsion angle needs to be converted into radians before the torsional stiffness can be calculated. Table 4 lists all of the calculation results. The stiffness results of Case 1 and Case 2 were nearly identical on account of the fact that the gap in the target bending stiffness design of the model was not large enough. No significant differences in bending stiffness were reflected in the results. It can be observed in Table 4 that the [±30/±25/±20/±20]s layup sequence had a more significant effect in improving the torsional stiffness, probably due to the fact that the torsional stiffness of Case 3 was greatest when the design target torsional stiffness was applied.

The predicted solution calculated by the model was close to the actual solution. The model, as far as we can tell, is generally feasible for material design. The ratio of the stiffness to fiber modulus in Table 4 can also indicate that the theoretical value is close to the actual test result. Case 2 was best for improving the flexural stiffness performance of the pipe at the same fiber modulus, while the laminate design used Case 3,which was best suited to improving the torsional stiffness properties of the pipe under the same fiber modulus. Therefore, we can see that lamination has a significant effect on the structural properties of the laminate.

According to the experimental results, the TGML model overpredicted the bending and torsional stiffness. This overprediction is due to the fact that the design parameters such as the angle and modulus of the model are theoretical values, but the actual specimen contains flaws. Simultaneously, the design of the loading experiment scheme affected the experimental results, however, the errors in the model were within the acceptable limits. Because of its accuracy and practicability, the TGML model can be used as a reference in engineering applications.

## 5. Conclusions

This paper proposed a method to accelerate the layup sequence design of composite laminates based on the TGML models. The basic idea of theoretical guidance is to preprocess the training data of the model through the CLT and Hashin theories so that the TGML models generate their output in a logical sequence. The data generated by the finite element model were validated by experiments and used to train the TGML models to solve the problem of composite material laminate design. Compared with traditional ML models without theory guides, TGML models have better regression performance, even with a small amount of data. In addition, TGML models have a significant advantage in terms of time efficiency compared with traditional FEM-based optimization iterations.

## Figures and Tables

**Table 1:** A comparison of the time spent by models on different training sets on the entire activity of the theory-guided model design framework.

Data	Process	Time (min)
2700 data	FEM	135
	Training—ML model	2
270 data	FEM	13.5
	Training—ML model	1.5
3000 data	GA	3

**Table 2:** The designs of the layups with optimum strength values under several load conditions.

Case No.	Load Conditions		Layups	Failure Index	
	Bending (N)	Torsion (N·m)		Bending (FEM)	Torsion (FEM)
1	1000	300	[±30/±30/±30/±25]s	0.473 (0.505)	0.493 (0.513)
2	600	300	[±35/±35/±50/±40]s	0.393 (0.361)	0.394 (0.392)
3	1000	150	[±20/±20/±20/±20]s	0.315 (0.345)	0.186 (0.178)

**Table 3:** The optimal layups and the modulus design under several stiffness requirements.

Case No.	Target Stiffness		Layups	*E*_1*M*_ (GPa)	ML Output Stiffness	
	Bending (N/mm)	Torsion (N·m/rad)			Bending (FEM) (N/mm)	Torsion (FEM) (N·m/rad)
1	250	1500	[±0/±35/±25/±15]s	266	250 (252)	1500 (1442)
2	300	1500	[±25/±10/±40/±5]s	312	300 (313)	1500 (1448)
3	200	2000	[±30/±25/±20/±20]s	281	200 (189)	2000 (1802)

**Table 4:** A comparison of the stiffness between the experiment and ML output under different layup sequences.

Case No.	Layups	Stiffness	Experiment	ML Output	Target Stiffness/*E*_1*T*_ (ML Output/*E*_1*T*_)
1	[±0/±35/±25/±15]s	Bending (N/mm)	99.3 ± 2.7	116	0.940 (1.087)
		Torsional (N·/rad)	628.7 ± 3.2	630.1	5.639 (5.530)
2	[±25/±10/±40/±5]s	Bending (N/mm)	97.1 ± 2.1	114.2	0.962 (0.993)
		Torsional (N·/rad)	634.5 ± 2.9	651.5	5.517 (5.665)
3	[±30/±25/±20/±20]s	Bending (N/mm)	98.7 ± 2.4	104.5	0.712 (0.909)
		Torsional (N·/rad)	788.6 ± 4.5	808.7	7.117 (7.032)

## Data Availability

Not applicable.
